# Editorial: Computational methods to analyze RNA data for human diseases

**DOI:** 10.3389/fgene.2023.1270334

**Published:** 2023-08-22

**Authors:** Pingjian Ding, Min Zeng, Rui Yin

**Affiliations:** ^1^ Center for Artificial Intelligence in Drug Discovery, School of Medicine, Case Western Reserve University, Cleveland, OH, United States; ^2^ School of Computer Science and Engineering, Central South University, Changsha, China; ^3^ Department of Health Outcomes and Biomedical Informatics, University of Florida, Gainesville, FL, United States

**Keywords:** RNA, miRNA, LnRNA, RNA virus, ceRNA network, machine learning/statistics, human disease

RNA, as a type of nucleic acid, forms one of the four fundamental macromolecules crucial for all known life forms. Unlike DNA (Deoxyribonucleic Acid), which typically serves as the primary genetic material in cells, many viruses use RNA as their genetic material. RNA viruses are known for their ability to mutate rapidly, and the emergence of novel strains and variants (Yin et al., 2020) is potentially responsible for a wide range of diseases, leading to epidemics or pandemics such as swine-origin flu pandemic (Yin et al., 2018) and COVID-19 (V’kovski et al., 2021; Yin et al., 2018; Ding and Xu, 2023). In addition, RNA plays critical roles in various biological processes, including gene expression, protein synthesis (Frye et al., 2018). Understanding the mechanisms and roles of RNA in disease pathogenesis and progression is crucial for advancing our knowledge of human biology and developing optimized therapeutic strategies to combat RNA-related diseases. Computational approaches like machine learning and statistics, have captured much attention in this field due to increasingly available diverse RNA datasets (Yin et al., 2022; Li et al., 2023; Yin et al., 2023). This Research Topic of Frontiers in Genetics features a Research Topic of the latest advances in applying and developing various kinds of computational methods to analyze RNA data towards non-coding RNAs (e.g., miRNA, lncRNA) and RNA viruses (e.g., influenza, coronavirus).

The ncRNAs are crucial for regulating gene expression at both the transcriptional and posttranscriptional levels within the transcriptome, without encoding proteins (Winkle et al., 2021). In particular, miRNAs are a type of small, single-stranded noncoding RNAs, about 19–25 nucleotides long, that have highly conserved sequences and can regulate gene expression at the post-transcriptional level. Through extensive research on miRNA in the context of development and disease, it has emerged as a compelling target for innovative therapeutic approaches (Shen et al., 2020a; Shen et al., 2020b; Li Peng et al., 2022). In this Research Topic, Luo et al. presented a comprehensive perspective of recent progress in miRNA-targeted therapeutics employing machine learning techniques. In addition to discussing resources and preprocessing of pharmacogenomic data, they also presented the main machine learning algorithms employed in identifying miRNA-disease associations. Given the limitations of current methods in constructing negative sample sets, Wei et al. introduced a clustering-based sampling approach called CSMDA to predict miRNA-disease associations. This method aims to address the Research Topic associated with negative sample selection in the context of miRNA-disease association prediction. Under a five-fold cross-validation, CSMDA computed an impressive Area Under the Curve (AUC) of 0.9610. Additionally, through validation with the dbDEMC database, it was confirmed that all predicted miRNAs, except hsa-mir-34c, were associated with colon cancer.

LncRNAs are a subset of ncRNAs characterized by their length, which exceeds 200 nucleotides. They have important functions in controlling gene expression at various levels, such as translational, transcriptional, and epigenetic processes (Qin et al., 2020). LncRNAs are crucial in controlling genes and proteins related to a range of human diseases like cancer (Xiao et al., 2018), digestive system Research Topic, and heart problems. Their role in disease regulation is well-established and holds promise for future therapies. Yao et al. proposed a computational model called GCHIRFLDA, which utilizes geometric complement heterogeneous information and random forest to predict lncRNA-disease associations. Under five-fold cross-validation, GCHIRFLDA achieved impressive performance metrics with an AUC of 0.9897 and an AUPR of 0.7040. The study demonstrated that 18 of the predicted lncRNAs were validated through records present in databases or published literature. Meanwhile, the presence of inherent sparsity in known heterogeneous bio-data poses a challenge for computational methods aiming to enhance the accuracy of prediction. Thus, Zhang et al. explored a novel multiple mechanisms to discover underlying lncRNA-disease associations (MM-LDA). By integrating the graph attention network (GAT) and inductive matrix completion (IMC), this approach boosts the prediction accuracy. Firstly, a multiple-operator aggregation was created as part of the n-heads attention mechanism in the GAT. Then, IMC was incorporated into the improved node feature, and subsequently, the LDA network underwent a reconstruction to address the cold start problem caused by insufficient data in either whole rows or columns of a known association matrix. Under 5-fold cross-validation, an AUC of 0.9395 and an AUPR of 0.8057 were computed. The results from MM-LDA suggested a potential link between HOTAIR and HTTAS and gastric cancer.

In recent years, there has been the proposal of a hypothesis about competing endogenous RNA (ceRNA) network (Salmena et al., 2011). Under this hypothesis, lncRNAs possess the capability to function as endogenous molecular sponges for miRNAs, indirectly regulating the expression of messenger RNAs (mRNAs). The intricate nature of the lncRNA-miRNA-mRNA network makes their dysregulation closely linked to the progression and onset of various human diseases. For example, Ye et al. (2019) discovered that the lncRNA MIAT increases the expression of CD47 by acting as a sponge for miR-149-5p, leading to the inhibition of efferocytosis in advanced atherosclerosis. Yang et al. (2021) conducted a study uncovering the role of lncRNA XIST as a ceRNA, promoting atherosclerosis by upregulating TLR4 expression through the mediation of miR-599. Additionally, they identified several putative ceRNA networks, including those associated with implantation failure (Feng et al., 2018), polycystic ovary syndrome (Ma et al., 2021), and epithelial ovarian cancer (Zhao et al., 2019). Chen et al. employed the CIBERSORT algorithm to investigate the potential ceRNA-related mechanism of Peripheral arterial occlusive disease (PAOD) and to identify the associated patterns of immune cell infiltration. They developed an immune-related core ceRNA network that offered valuable insights into the molecular mechanisms underlying Peripheral Arterial Occlusive Disease (PAOD). This network consisting of CREB1, LINC00221, miR-20b-5p, and miR-17-5p, along with the infiltrating immune cells, specifically M1 macrophages and monocytes. Luo et al. introduced a lncRNA–mRNA network based on POI (POILMN) to identify essential lncRNAs. This research yielded a Research Topic of 288 differentially expressed mRNAs and 244 differentially expressed lncRNA. Ultimately, Through the application of topological analysis, POILMN identified four intersecting lncRNAs based on two centralities, namely, degree and betweenness.

CircRNA is a class of ncRNAs that forms a covalently closed loop structures (Li et al., 2020; Xiao et al., 2020; Peng et al., 2022; Peng et al., 2023). CircRNA molecules have been observed or artificially synthesized in various organisms, including mammals (Xu and Zhang, 2021) and viruses (Tan and Lim, 2021). The interactions between miRNAs and circRNAs have been demonstrated to modify gene expression and play a regulatory role in diseases. Therefore, He et al. introduced a novel approach called GCNCMI, which utilizes a graph convolutional neural (GCN) network to uncover latent associations between miRNAs and circRNAs. GCNCMI initially examines the underlying connections between neighboring nodes in the GCN network. Afterward, it iteratively spreads this connection information across the graph convolutional layers. Lastly, the embeddings produced by each layer were combined to output the ultimate prediction results. GCNCMI achieved an AUC of 0.9312 and an AUPR of 0.9412. The results from GCNCMI showed that 8 interactions involving hsa-miR-149-5p and 7 interactions involving hsa-miR-622 were validated.

Additionally, mitochondrial dysfunction could be among the molecular mechanisms implicated in obstructive sleep apnea (OSA) and its concurrent conditions. Despite several studies reporting the involvement of various proteins and miRNAs in OSA (Targa et al., 2020; Pinilla et al., 2021), the impact of OSA on genes and pathways, particularly concerning mitochondrial dysfunction, remains largely unexplored. In a previous study by Li et al. (2017), differentially expressed miRNAs were reported in OSA, but their specific association with mitochondrial dysfunction was not established. Liu et al.developed a novel diagnostic model consisting of a four-gene signature related to mitochondrial dysfunction. Using gene expression related to mitochondrial dysfunction, all samples were categorized into two clusters, with an additional subdivision of three clusters identified specifically among the samples with OSA. In the OSA samples compared to control samples, Significant differences were noted in the levels of M0 and M1 macrophages as well as plasma cells. Additionally, within the clusters associated with mitochondrial dysfunction in OSA samples, various immune cell types, particularly T cells, showed significant differences.

Although multiple databases offer information on virus-host protein interactions, they often lack detailed information about strain-specific virulence factors or the specific protein domains implicated in the interactions (Yin et al., 2017; Yin et al., 2021). Several databases may have incomplete representation coverage of influenza strains of influenza strains due to the challenge of sifting through extensive literature to gather comprehensive information. No existing database has provided complete records of strain-specific protein-protein interactions for all types of Influenza A viruses. In particular, Ng et al. presented an innovative network that predicts domain-domain interactions between proteins from the mouse host and influenza A virus (IAV). By incorporating vital virulence details like lethal dose, this network facilitates a methodical exploration of disease factors. They created a network of interacting protein domains from both mouse and viral proteins, representing them as nodes and using weighted edges to show their interactions.

In summary, this Research Topic centers on the recent progress in utilizing and refining diverse computational methods, including machine learning and statistical techniques, to analyze RNA data related to RNA viruses and non-coding RNA. As a result, these analyses have delved into the biological disease mechanisms and aided in the understanding of human diseases, leading to improved preventive measures, diagnoses, and treatments.
